# “What Does Weight Have to Do with It?” Parent Perceptions of Weight and Pain in a Pediatric Chronic Pain Population

**DOI:** 10.3390/children3040029

**Published:** 2016-11-14

**Authors:** Keri R. Hainsworth, Kristen E. Jastrowski Mano, Alison M. Stoner, Kim Anderson Khan, Renee J. Ladwig, W. Hobart Davies, Ellen K. Defenderfer, Steven J. Weisman

**Affiliations:** 1 Department of Anesthesiology, Medical College of Wisconsin, Milwaukee, WI 53226, USA; kanderson@chw.org (K.A.K.); sweisman@chw.org (S.J.W.); 2 Department of Psychology, University of Cincinnati, Cincinnati, OH 45221, USA; manokn@ucmail.uc.edu; 3 Department of Psychiatry, University of Massachusetts Medical School, Worcester, MA 01655; USA; alison.stoner@umassmemorial.org; 4 Jane B. Pettit Pain and Headache Center, Children’s Hospital of Wisconsin, Milwaukee, WI 53226, USA; rladwig@chw.org; 5 Department of Psychology, University of Wisconsin-Milwaukee, Milwaukee, WI 53201, USA; hobart@uwm.edu (W.H.D.); defende2@uwm.edu (E.K.D.)

**Keywords:** chronic pain, obesity, pediatrics, parents, attitudes

## Abstract

Tailored pain management strategies are urgently needed for youth with co-occurring chronic pain and obesity; however, prior to developing such strategies, we need to understand parent perspectives on weight in the context of pediatric chronic pain. Participants in this study included 233 parents of patients presenting to a multidisciplinary pediatric chronic pain clinic. Parents completed a brief survey prior to their child’s initial appointment; questions addressed parents’ perceptions of their child’s weight, and their perceptions of multiple aspects of the relationship between their child’s weight and chronic pain. The majority (64%) of parents of youth with obesity accurately rated their child’s weight; this group of parents was also more concerned (*p* < 0.05) about their child’s weight than parents of youth with a healthy weight. However, the majority of parents of youth with obesity did not think their child’s weight contributed to his/her pain, or that weight was relevant to their child’s pain or pain treatment. Overall, only half of all parents saw discussions of weight, nutrition, and physical activity as important to treating their child’s pain. Results support the need for addressing parents’ perceptions of their child’s weight status, and educating parents about the relationship between excessive weight and chronic pain.

## 1. Introduction

Currently, 33% of youth in the U.S.A. are overweight or obese [1] and there is evidence to suggest that the prevalence of youth with elevated weight may be greater in chronic pain populations than in normative samples [2]. Additionally, there is increasing evidence that obesity exacerbates the impact of chronic pain in children. The first study to provide such evidence found that children and adolescents with co-occurring chronic pain and obesity reported lower health-related quality of life in all domains of functioning (i.e., physical, emotional, social, and school functioning) than youth who are overweight or those with healthy weight [3]. This study also showed that youth with chronic pain and obesity are 2.3 times more likely to report impaired physical functioning than youth with chronic pain alone and more than six times more likely than youth with obesity alone. Wilson and colleagues [2] found that in a sample of youth with chronic pain, body mass index (BMI) predicted functional disability for rigorous activities, based on parent reports. In a study focused on pediatric headache, Hershey et al. [4] found an association between obesity and both headache frequency and disability. This study also showed that for patients with weight concerns, weight loss was associated with reduced headache frequency at three- and six-month follow-up points. Most recently, Stoner et al. [5] found that obesity impedes improvement in functional disability, an important patient-reported outcome [6,7]. Among patients in active treatment for chronic pain, those with a healthy weight showed significant improvement in functional disability over time, in contrast to youth with both morbidities whose level of functional disability remained stagnant [5].

The studies above demonstrate the impact that obesity has on pediatric chronic pain. It is particularly concerning that obesity appears to impede improvement despite multidisciplinary pain treatment. However, to our knowledge, no tailored interventions exist to meet the needs of youth with these dual conditions. Therefore, it is critical that we go beyond the initial call to simply screen for obesity in pediatric chronic pain [3] and begin to develop tailored treatment approaches to meet the needs of this population. If we do *not* address weight in youth suffering from chronic pain in childhood, it is likely that these children may face a lifetime of suffering. In a qualitative study focused on adults with chronic pain and obesity, participants poignantly describe the cycle of pain and disability that ensues from the co-morbid state. In contrast, pediatric chronic pain patients may experience several important benefits if we *do* address their weight in addition to their chronic pain. For example, by increasing physical activity, we may reduce pain through decreased levels of systemic inflammation, elevations of mood, and decreased biomechanical forces on the back and lower extremities [8].

Before such approaches can be developed and implemented, however, we need to know more about factors that may potentially hinder the acceptance of treatment strategies that target both pain and weight. Looking to the pediatric obesity literature, one likely example is parents’ perceptions of their child’s weight; parents commonly misperceive their child’s weight [9,10,11,12,13], which in turn has important clinical implications. Taveras et al. [14] found that parents who misperceived their child’s elevated weight were more likely to refuse their child’s participation in a weight management program than parents who accurately rated their child’s weight. If parents of youth with chronic pain misperceive their child’s weight, they may be less likely to adhere to treatment recommendations that involve physical activity or other strategies that might be construed as weight-related recommendations.

This study sought to characterize parents’ perceptions of and attitudes around weight in the context of pediatric chronic pain. The primary aims of this study were to determine whether parents of youth presenting to a multidisciplinary pain clinic with co-occurring chronic pain and weight concerns (1) are accurate in their assessment of their child’s weight; and (2) saw their child’s weight as contributing to his/her pain. We also aimed to (3) assess whether parents of children in all weight groups thought that discussions of weight, nutrition and physical activity might be important to treating chronic pain, particularly among parents of youth with co-morbid weight concerns. Secondary aims were to assess parents’ level of concern about their child’s weight, as well as factors that may influence adherence to integrated pain and weight interventions.

## 2. Methods

### 2.1. Participants

This study was part of a quality improvement (QI) project aimed at developing pain treatment approaches for our patients with co-morbid obesity. The convenience sample included consecutive parents of patients seen in a multidisciplinary pediatric pain clinic, over a designated 4.5 month timeframe.

### 2.2. Procedures

Upon arrival for their child’s initial appointment, parents were asked to complete a brief survey along with other paperwork. The hospital Institutional Review Board approved a retrospective chart review and use of the data for study purposes.

### 2.3. Measures

#### 2.3.1. Demographic Data

Demographic data were obtained from the electronic medical record (EMR), and included patient age, gender, race, ethnicity, weight, height, BMI and BMI percentile. Height and weight are measured by nurses at the time of the initial appointment, and directly entered into the EMR. Both BMI and BMI percentile are calculated within the EMR by the software program (EPIC), and are based on the Centers for Disease Control and Prevention (CDC) profiles for gender and age adjusted weight scales [15]. The American Academy of Pediatrics [16] criteria were used to categorize patients’ weight status: <5th percentile = underweight; ≥5th percentile to <85th percentile = healthy weight; ≥85th percentile to <95th percentile = overweight; ≥95th percentile = obese.

#### 2.3.2. Survey Questions

The survey used in this study was created for the QI project by a panel of experts in pediatric chronic pain. To minimize completion time in the waiting room, the survey was intentionally designed to be brief, consisting of only five items. Survey questions are listed here.

1. Question 1, “*Do you consider your child to be*” was presented with the response options “Overweight,” “Underweight,” “Just about the right weight,” and “Don’t know.” This question was adopted and modified slightly from a study aimed at examining the accuracy of maternal perceptions of their child’s weight status, in a group of Hispanic WIC (a nutritional program for Women, Infants, and Children) participants [10].
2. Question 2, “*On a scale of 1*–*5, how concerned are you about your child*’*s present weight or body size?*” was adopted verbatim from a study by Campbell et al. [9]. Response options ranged from “Not Concerned” (1) to “Very Concerned” (5). This question was chosen to address the first of three primary aims.
3. Question 3, “*Do you think that your child*’*s weight contributes to your child*’*s pain?*” was created to address the second of three primary aims. Response options were “yes” or “no.”
4. Question 4, “*Do you believe that discussions of weight, nutrition and physical activity might be important to treating pain?*” was created to address the third of three primary aims. Response options were “yes” or “no.”
5. Item 5, which asked parents to “*Check all that apply*”, was designed to capture factors that may affect adherence to integrated chronic pain and weight interventions. The five response options included (a) “I do not think weight is relevant to his/her pain treatment”; (b) “Weight has been discussed with healthcare providers several times”; (c) “We have tried to address weight in the past without success”; (d) “I have little hope he/she will be able to lose weight”; and (e) “My child’s pain prevents him/her from losing weight”. Reponses were treated as endorsed or not endorsed. Item b specified “several times,” in an attempt to get at more than just a cursory mention of weight by any provider in the past.

### 2.4. Data Analysis

Descriptive statistics were used to characterize the children of the participants (i.e., the pediatric pain patients) and parental responses to survey questions. Parametric (Student’s *t*-tests, and analysis of variance (ANOVA)) statistics were used for continuous variables and non-parametric (chi-square) statistics were used for categorical variables. To determine accuracy of parental estimates of their child’s weight, any response (including “I don’t know”) that did not match the child’s measured weight status was categorized as inaccurate.

## 3. Results

### 3.1. Patient Population

A total of 239 parents completed surveys. No information is known about parents who did not complete surveys or were missed. Only six of the participants’ children were underweight, and therefore these surveys were excluded from analyses, yielding a final sample size of 233 parents. Patients (their children) were primarily adolescent females (64%, mean (*M*) = 13 ± 3.3 years; 4 to 18 years). Patients were also primarily Caucasian (*n* = 179, 78%), with 15% (*n* = 35) African American, and were primarily non-Hispanic (*n* = 197, 86%). The majority of patients were classified as having a healthy weight (57% of the sample), with 18% classified as overweight, and 25% as obese. The vast majority of the sample (*n* = 169, 73%) was being treated for a primary pain location of the head, followed equally by back (*n* = 19, 8%), abdomen (*n* = 19, 8%), and extremity pain (*n* = 19, 8%). Demographics are presented in Table 1. No differences across weight groups were found for any of the demographic variables (including pain location; data not shown).

### 3.2. Parents’ Assessment of Their Child’s Weight Status

The data from question 1, addressing the accuracy of parental perceptions of their child’s weight, are shown in Table 2. As indicated, any response (including “I don’t know”) that did not match the child’s measured weight status was categorized as inaccurate. Results suggest that the majority (64%) of parents whose children are obese accurately rate their child’s weight status, with about one-quarter (27%) rating the child’s weight as “just about the right weight”. This pattern reflects greater accuracy than reports by parents from other populations [10,11]. In contrast, the majority (90.5%) of parents whose children are overweight misperceive their child’s weight status, which is very similar to reports of parents in the pediatric obesity literature [10].

### 3.3. Parental Concern about Their Child’s Present Weight or Body Size

Parents of obese chronic pain patients were significantly (*p* < 0.001) more concerned (*M* = 2.7 ± 1.4) about their child’s weight than parents of youth with overweight status (*M* = 1.4 ± 0.8) or parents of youth with healthy weight (*M* = 1.4 ± 0.9). However, the average rating from parents of youth with obesity showed only moderate concern, and was only slightly higher than the average rating from the latter two subgroups. Further examination of responses by parents of obese youth shows that while approximately one-third (33%, *n* = 18/55) indicated that they were very concerned (endorsement of response options 4 or 5) about their child’s weight, the majority, about one half (47%, *n* = 26/55), were not concerned (endorsement of response options 1 or 2). Using the same question, Campbell reported that the majority of parents whose overweight and obese children (3 to 17 years old) were referred to a multidisciplinary weight management clinic were very concerned about their child’s weight, with 77% rating their concern as a “5” (very concerned). In the current sample, only 12.7% (*n* = 5) of the parents with obese children rated their concern as a “5”. A single-sample *t*-test using Campbell’s data [9] indicates that parents of youth with both chronic pain and obesity (*M* = 2.7 ± 1.4) are less concerned (*p* < 0.05) about their child’s weight than parents whose children have obesity alone (*M* = 4.7 ± 0.7).

### 3.4. Parental Perception of a Weight-Pain Association

In response to question 3 (data for questions 3 and 4 are shown in Table 3), asking whether their child’s weight contributed to their child’s pain, the vast majority of the whole sample responded “no”. However, significant (*p* < 0.001) weight-based differences were found in the responses to this question. All (100%) parents of overweight children and 76% (40/53) of parents with obese children, thought that their child’s weight did not contribute to the child’s pain. In fact, only 24.5% of the parents with obese children responded “yes” to this question. To examine these responses more closely, we conducted a second chi-square analysis to determine if parents’ responses to this question were related to the accuracy of the estimate of their child’s weight. The analysis showed that parents who accurately rated their obese child as overweight were more likely to respond “yes” to this question (*χ^2^* = 16.88 (2), *p* < 0.001; *Standardized Residual* [*Std. Resid]* 2.9). This subset was approximately one-third of the parents who accurately rated their obese child’s weight (*n* = 12).

### 3.5. Parental Responses to the Question *“Do You Believe That Discussions of Weight, Nutrition and Physical Activity Might Be Important to Treating Pain?”*

Just over half (56%) of the entire sample (i.e., parents of youth in all weight categories) endorsed “no” in response to question 4. In fact, there were no weight group differences in response to this question (*p* > 0.05), with approximately half of the parents in each weight group indicating that these discussions are not important to treating pain. Four parents wrote in “maybe” in response to this question (responses not included in analysis). Two of the children represented by these latter responses were obese, one was overweight, and one was a healthy weight.

### 3.6. Parents’ Endorsement of Factors That May Act as Barriers to Integrated Chronic Pain and Weight Interventions

#### 3.6.1. I Do Not Think Weight Is Relevant to His/Her Pain or Pain Treatment.

The majority of the sample (83%) endorsed this item, indicating their perception that weight is not relevant to their child’s pain treatment (data for question 5a–e are shown in Table 4). Consistent with this pattern of results, approximately 88% of parents of overweight youth and 68% of parents of obese youth endorsed this item. Parents of obese youth were over-represented in the unendorsed group (*p* < 0.001; *Std. Resid*. 2.7), suggesting that a small subset of parents with obese children may think that weight is relevant to their child’s pain.

#### 3.6.2. Weight Has Been Discussed with Healthcare Providers Several Times

Across the entire sample, only 13% of parents endorsed the item “weight has been discussed with healthcare providers several times”. Though plausible that parents of obese youth would endorse this option more frequently than parents of overweight or healthy-weight youth, this was not the case (*p* > 0.05). Of parents with obese children, only 21% indicated that weight had been discussed several times with healthcare providers.

#### 3.6.3. We Have Tried to Address Weight in the Past without Success

Across the sample, only 6% of parents indicated that they had tried to address their child’s weight without success. The weight-based analysis showed that significantly more parents of obese youth endorsed previous, unsuccessful attempts to address weight (*p* < 0.01); however, this only represented 16% (9/56) of the obese subgroup.

#### 3.6.4. I Have Little Hope He/She Will Be Able to Lose Weight

No weight group differences were found for this item (*p* > 0.05), as only a few parents endorsed it: one parent of a child with a healthy weight (0.8%), and three parents of children with obesity (5.4%) indicated that they have little hope that their child will be able to lose weight.

#### 3.6.5. My Child’s Pain Prevents Him/Her from Losing Weight

Across the entire sample, only 5% of parents endorsed this item. Despite the small number overall, the analysis of weight-based differences showed that significantly more (*p* < 0.001) parents of obese youth (16%) endorsed this item compared to parents of either overweight or healthy-weight youth.

## 4. Discussion

The goal of this study was to better understand parents’ attitudes and perceptions about weight in the context of pediatric chronic pain. The results showed that among parents whose children have both chronic pain and obesity, more than half accurately rate their child’s weight status. However, as a group, the majority were not concerned about their child’s weight, did not see a relationship between their child’s pain and weight, did not see weight as relevant to their child’s pain, and did not see discussions of weight, nutrition and physical activity as important to treating pain. In short, for most parents whose children have co-occurring chronic pain and obesity, there is little foundation upon which to build a case to treat both weight and pain simultaneously.

We hypothesized that having a child with a medical condition (i.e., chronic pain) might improve the accuracy of parents’ perceptions of their child’s weight. It is common for youth with chronic pain to be seen by multiple practitioners prior to being seen in a pain clinic [17]. Therefore, it is plausible that having repeated weight/height measurements increases awareness of their child’s weight status, and in turn, the accuracy of their estimates. The fact that 64% of parents of obese youth accurately estimated their child’s weight status, a rate that is higher than reports by parents of youth with obesity alone, provides support for this hypothesis. Nonetheless, it is concerning that 36% of parents of obese children and 91% of parents of overweight children inaccurately estimated their child’s weight status. Taveras et al. [14] found that a parent’s misperception of their child’s weight status was associated with not allowing their child to participate in a weight management program. Given that almost half of the children represented in this sample were overweight/obese, it is particularly concerning that collectively, 59% of their parents inaccurately rated their weight status. Furthermore, we found that when parents of chronic pain patients with obesity accurately estimated their child’s weight status, they were also more likely to say that their child’s weight contributes to the child’s pain. Although speculative, if the results of Taveras et al. translate to the pediatric pain population, patients who would benefit from tailored interventions could miss out on attempts to treat weight in the context of pediatric pain, and/or may be less likely to adhere to treatment recommendations that involve physical activity or what might be perceived as weight-related recommendations. Based on the influential role of parents’ attitudes and behaviors on their child’s own health behaviors, Duncan [12] has called for interventions to correct parents’ misperceptions of their child’s weight. This type of parent education could certainly be part of tailored interventions for chronic pain and weight, and could be as simple as pain practitioners pointing out that the child is overweight/obese and providing some examples of the ways in which weight and pain are related. Based on the fact that nearly all parents of youth with overweight status inaccurately rated their child’s weight, interventions must include parents of children with overweight, as well as those whose children have obesity.

Other results from this study suggest that parents lack awareness of the relationship between weight and chronic pain. In their review of the literature on pain and obesity, Narouze and Souzdalnitski [18] indicate that the relationship between obesity and chronic pain has been a focus of attention since the late 1990s, and that the evidence points to a strong association between these chronic conditions. The evidence provided in their review of high-quality studies demonstrates a relationship between obesity and pain in weight-bearing joints, but also between obesity and headache/migraine pain, upper extremity pain, chronic widespread pain and fibromyalgia, abdominal/pelvic pain and chronic neuropathic pain. Despite this evidence, as well as a worldwide focus on obesity in adults and children, our data suggests that parents of both overweight and obese children are not aware that these conditions are related. Parents of overweight and obese children did not think that their child’s weight contributes to their child’s pain, nor did they think that weight was relevant to the child’s pain treatment or that discussions of weight, nutrition and physical activity might be important in treating pain. Several factors may shed light on these results. First, these results may be explained in part by the fact that only 7% of parents with overweight children and 21% of parents with obese children reported discussing weight with healthcare providers. This may suggest that providers lack an awareness of the relationship between obesity and chronic pain, and/or that they lack training in counseling patients with both conditions. Patients may have had up to 15 office visits for their chronic pain in the year prior to being seen in a pain center [17]; therefore, it is surprising that so few had been discussing weight concerns with providers. To the degree that providers are not talking to patients and their families about these concerns, we can understand why parents may not be aware of the relationship. It is noteworthy that multidisciplinary chronic pain management programs commonly include approaches to increase activity [19]. Additionally, it is not uncommon for weight gain to follow injury and pain in childhood [20], and some pain medications (e.g., pregabalin and gabapentin) are associated with weight gain [18]. Therefore, educating parents about the importance of weight, nutrition and physical activity is important, regardless of the weight status of the patient at the time of intake. Additionally, these results may be explained in part by the type and location of patient pain complaints. It is important to note that almost 73% of the children represented in this study reported a primary pain location of the head. While it may/may not be more intuitive for parents to be aware of a link between obesity and, for example, lower back pain, it would be less intuitive to be aware of a link between obesity and chronic headache or migraine pain. Studies involving adults (e.g., [21,22]) and children [4] have provided strong evidence of a link between these two conditions, which underscores the necessity for further parent education.

This study also aimed to better understand factors that may act as barriers to the acceptance of future tailored interventions for children with co-occurring chronic pain and weight concerns. Overall, very few parents of overweight or obese patients indicated that they had previous unsuccessful attempts to lose weight, had little hope that their child would lose weight, or that their child’s weight prevented him/her from losing weight. This pattern of responses is consistent with a lack of awareness of a chronic pain/obesity relationship, and suggests that these may not actually be common barriers affecting the acceptance of integrated chronic pain and weight interventions in this population. However, it is plausible that attempts to increase awareness of the child’s overweight/obesity status may alter parents’ responses to these questions, and/or may increase worry over their child’s weight, in turn affecting adherence to treatment recommendations and/or readiness to change. If parents of youth with chronic pain do not expect their child’s pain provider to address their child’s weight concerns, what seemed like a simple matter of educating parents to raise awareness may escalate into a need for additional treatment components to assuage worry. In this regard, the sensitivity of the provider’s approach may be particularly important in tailored interventions. Furthermore, it is well known that having a child with a chronic pain condition is stressful, and is associated with reduced parental health-related quality of life [23] and numerous direct and indirect burdens, such as high financial costs, time spent in appointments and missed time at work [24,25]. It is plausible that parents are compartmentalizing their child’s chronic pain and weight conditions, and therefore pointing out that they should be concerned about their child’s weight in addition to his/her chronic pain might increase worry, and in turn decrease acceptance and adherence to integrated weight and chronic pain interventions. If this is the case, pain providers can play a critical role by helping to shift parents’ perspective from seeing chronic pain and obesity as two separate issues into understanding how pain and weight are inherently intertwined.

Based on the current and previous [2,3,5] findings, it is critical that we go beyond the initial call to simply screen for obesity in pediatric chronic pain [3], and begin to develop tailored treatment approaches to meet the needs of this population. *Not addressing* [26] weight in children with chronic pain may have devastating lifetime consequences, while *addressing* [8] weight in this population has the potential to reduce pain and improve functioning. In fact, Narouze and Souzdalnitski [18,27] suggest that the infrastructure to treat both chronic pain and obesity already exists in pain medicine, including medications, physical and psychological rehabilitation, and interventional management. Although these authors provide potentially effective options for the co-management of both conditions, as well as nuances that require consideration for each individual condition, the majority of the research upon which these recommendations have been based has involved adults with co-occurring chronic pain and obesity. These recommendations should be empirically evaluated in a pediatric population. However, we do agree that “further research should focus on comparing existing, and developing new, evidence-based strategies for the treatment of these complex patients, and exploring the advantages of simultaneously managing obesity and chronic pain” [27] (p. 219).

There are several limitations to this study. Similar to the limitations outlined by Connelly et al. [28], parents in the current study completed the surveys in a naturalistic setting, which may have affected data completion and the response rate, and the question set was minimized in order to limit interruption to the clinical flow. While this method allows for broad characterizations, it precludes the depth of inquiry required to fully understand parent perceptions and attitudes. Questions 2 and 3 were taken from studies for which no psychometric data are available. Related to this limitation, it is possible that the pattern of responses was affected by the forced choice response options and/or the phrasing of the questions. For example, responses to questions 3 and 4 may have been affected by the yes/no response choices. Additionally, responses to the question regarding discussions of weight, pain, and physical activity may have been different if the three options had been presented in separate questions. While this is improbable, based on the overall pattern of responses, without follow-up questions, it is not possible to determine whether responses would have been different. In addition, the sample was comprised of parents of primarily adolescent, Caucasian female patients. While this is common for most pediatric chronic pain samples [19], the demographic make-up limits the generalizability of the findings. Finally, it is clear that acceptance and adherence to future tailored interventions will be affected by a number of factors, including individual and contextual factors (see Simons et al. [19]). Among such factors are the patient’s own perceptions and attitudes about weight in the context of chronic pain. Future studies should not only examine parental attitudes in greater depth, but should also examine patient attitudes. Such studies should examine if and to what extent attitudes and perceptions interact with other factors to affect parent and patient acceptance of chronic pain interventions that also target weight.

Overall, the results of this study suggest that two salient factors characterize parental perceptions and attitudes about weight in the context of pediatric chronic pain. First, accurate estimates of weight status do not translate into an appreciation of the complex role that weight may play in an overweight or obese child’s chronic pain condition. Second, parents of children with chronic pain and overweight/obesity did not report expected barriers to acceptance of, or adherence to, tailored interventions. Specifically, most did not endorse failed attempts to reduce their child’s weight, a sense of hopelessness about their child losing weight, or the belief that their child’s pain prevents him/her from losing weight. Based on our findings, we offer two primary recommendations prior to implementing tailored interventions in the context of pediatric chronic pain and obesity: (1) pain providers should address parental misperceptions of their child’s weight; and (2) the integration of weight management into chronic pain interventions should begin with educating parents about the relationships between weight and pain.

## Figures and Tables

**Table 1:** Patient demographics. Categorical variables are listed as *n* (%); continuous variables are listed as mean (standard deviation, SD).

		Total Sample (233)	Healthy Weight (133, 57.1%)	Overweight (42, 18.0%)	Obese (58, 24.9%)
Gender ^1^	Male	83 (35.6)	46 (34.6)	16 (38.1)	21 (36.2)
	Female	149 (63.9)	87 (65.4)	26 (61.9)	36 (62.1)
Age		13.00 (3.30)	12.57 (3.35)	13.57 (2.81)	13.59 (3.38)
Race ^1^	African American	35 (15.6)	23 (18.0)	4 (9.8)	8 (14.5)
	Caucasian	174 (77.7)	98 (76.6)	35 (85.4)	41 (74.5)
	Multi-racial	6 (2.7)	1 (0.8)	1 (2.4)	4 (7.3)
	Other	9 (4.0)	6 (4.7)	1 (2.4)	2 (3.6)
Ethnicity ^1^	Hispanic	33 (14.3)	18 (13.7)	3 (7.1)	12 (21.1)
	Non-Hispanic	197 (85.7)	113 (86.3)	39 (92.9)	45 (78.9)

^1^ Missing cases = one gender, nine race, three ethnicity; no differences across weight groups for any variable (all *p* > 0.05).

**Table 2:** Parental responses to question 1 shown in relation to the patient’s measured weight status [10].

Measured Weight Status				
Parent Perceptions ^1^	Healthy Weight	Overweight	Obese	Total
Overweight	1 (0.8)	**4 (9.5)**	**36 (64.3)**	41 (18.0)
Underweight	11 (8.5)	1 (2.4)	0 (0)	12 (5.3)
Just about the right weight	**117 (90)**	35 (83.3)	15 (26.8)	167 (73.2)
Don’t know	1 (0.8)	2 (4.8)	5 (8.9)	8 (3.5)

^1^ *n* = 228 (five missing cases). Boldface values indicate accuracy in parental perceptions of their child’s weight.

**Table 3:** Parental responses to questions 3 and 4 shown in relation to the patient’s measured weight status. Data reflect *n* (%) of “no” responses.

Survey Question	Total Sample	Healthy Weight	Overweight	Obese	*p*-Value
Q3. Do you think that your child’s weight contributes to your child’s pain?	196 (89.1)	115 (91.3)	41 (100) ^1^	40 (75.5) ^2^	<0.001
Q4. Do you believe that discussions of weight, nutrition and physical activity might be important to treating pain? ^3^	116 (55.5)	73 (60.3)	19 (50.0)	24 (48.0)	>0.05

^1^ Parents of overweight youth were under-represented in the group responding yes to this question (*Std. Resid*. -2.1); ^2^ Parents of obese youth were over-represented in the group responding yes to this question (*Std. Resid*. 3.0); ^3^ Four parents who wrote in “maybe” were excluded from this analysis.

**Table 4:** Parental responses to question 5a–e shown in relation to the patient’s measured weight status. Data reflect *n* (%) of parents who endorsed each option.

Survey Question	Total Sample	Healthy Weight	Overweight	Obese	*p*-Value
Q5a. I do not think weight is relevant to his/her pain or pain treatment	189 (82.9)	115 (87.8)	36 (87.8)	38 (67.9) ^1^	<0.001
Q5b. Weight has been discussed with healthcare providers several times	29 (12.7)	14 (10.7)	3 (7.3)	12 (21.4)	>0.05
Q5c. We have tried to address weight in the past without success	14 (6.1)	4 (3.1)	1 (2.4)	9 (16.1) ^2^	<0.01
Q5d. I have little hope he/she will be able to lose weight	4 (1.8)	1 (0.8)	0 (0)	3 (5.4)	>0.05
Q5e. Child’s pain prevents him/her from losing weight	11 (4.8)	2 (1.5)	0 (0)	9 (16.1) ^3^	<0.001

^1^ Parents of obese youth were over-represented in the unendorsed group (*Std. Resid*. 2.7); ^2^ Parents of obese youth were over-represented in the endorsed group (*Std. Resid*. 3.0); ^3^ Parent of obese youth were over-represented in the endorsed group (*Std. Resid*. 3.8).
